# Correlative Gene Expression to Protective Seroconversion in Rift Valley Fever Vaccinates

**DOI:** 10.1371/journal.pone.0147027

**Published:** 2016-01-19

**Authors:** Richard C. Laughlin, Kenneth L. Drake, John C. Morrill, L. Garry Adams

**Affiliations:** 1 Department of Veterinary Pathobiology, Texas A&M University, College Station, TX 77843, United States of America; 2 Seralogix LLC, 335 Bee Cave Rd, Suite 607, Austin, TX 78746, United States of America; 3 Department of Microbiology and Immunology, University of Texas Medical Branch, Galveston, TX 77555, United States of America; George Mason University, UNITED STATES

## Abstract

Rift Valley fever Virus (RVFV), a negative-stranded RNA virus, is the etiological agent of the vector-borne zoonotic disease, Rift Valley fever (RVF). In both humans and livestock, protective immunity can be achieved through vaccination. Earlier and more recent vaccine trials in cattle and sheep demonstrated a strong neutralizing antibody and total IgG response induced by the RVF vaccine, authentic recombinant MP-12 (arMP-12). From previous work, protective immunity in sheep and cattle vaccinates normally occurs from 7 to 21 days after inoculation with arMP-12. While the serology and protective response induced by arMP-12 has been studied, little attention has been paid to the underlying molecular and genetic events occurring prior to the serologic immune response. To address this, we isolated RNA from whole blood of vaccinated calves over a time course of 21 days before and after vaccination with arMP-12. The time course RNAs were sequenced by RNASeq and bioinformatically analyzed. Our results revealed time-dependent activation or repression of numerous gene ontologies and pathways related to the vaccine induced immune response and its regulation. Additional bioinformatic analyses identified a correlative relationship between specific host immune response genes and protective immunity prior to the detection of protective serum neutralizing antibody responses. These results contribute an important proof of concept for identifying molecular and genetic components underlying the immune response to RVF vaccination and protection prior to serologic detection.

## Introduction

Rift Valley fever Virus (RVFV) (family Bunyaviridae, genus Phlebovirus) is a segmented, negative-stranded RNA virus and the causative agent of the vector-borne zoonotic disease, Rift Valley fever (RVF). The initial outbreak of RVF occurred in 1931 in the Rift Valley of Kenya in sheep, cattle and humans [1]. Currently, RVFV is considered endemic across Africa from Mauritania and Senegal in the west, Mozambique, South Africa and Namibia in the south, and north into Egypt and the Sinai peninsula [2]. The spread outside the African continent was likely related to trade of livestock and presence of competent vectors [3, 4]. Implicit to the transmission of RVFV are three genuses of mosquitos, *Aedes*, *Culex*, and *Anopheles* [5–7]. Importantly, the natural range of at least one of these vectors, *Aedes*, has expanded from tropical regions in south China and Indian Ocean Islands to other tropical and temperate zones in all continents, due to the passive transport of viable eggs and subsequent adaptation (reviewed in [8]). In addition, alterations to the global climate, as well as local forestation, development and biodiversity, likely to play significant roles in expanded vector range, habitat and density [9, 10]. RVFV outbreaks have had significant health, social and economic consequences, requiring considerable expenditure of resources to interrupt the infectious cycle, monitor susceptible populations and treat exposed individuals. An outbreak of RVF in a developed country or densely populated (people or livestock) region would be expected to have major health, economic and social impacts, as livestock movement would be severely limited, and affected animals quarantined or culled, and human populations would have high morbidity with variable levels of mortality [8, 11].

While susceptibility of naïve individuals to RVFV infection is high, protection can be achieved by humoral immune responses or colostrum [12, 13]. In an outbreak scenario, great energy, fiscal resources as well as human and animal lives could be saved with the single administration of a vaccine that elicits rapid humoral responses and a long-term protective immunity. Several vaccines have been developed to achieve this goal, including recombinant RVFV protein vaccines, formalin-inactivated vaccines NDBR-103 and TSI-GSD-200 strains as well as live-attenuated versions MP-12, Clone13 and various derivatives (reviewed in [14]). In our previous work, we reported a rapid immune response with high serum neutralizing antibody titers as measured by the plaque reduction neutralizing test (PRNT_80_), and a strong long-term immune response measured by a RVFV antigen-specific IgG enzyme-linked immunosorbent assay (ELISA) induced by authentic recombinant MP-12 (arMP-12) in both pregnant sheep and cattle [15–17]. In 2014, arMP-12-NSm21/384 vaccinated sheep demonstrated excellent protection against development of RVFV viremia and fever after a single dose when challenged with wild type ZH501 RVFV four weeks post vaccination [18, 19].

Recent studies assessing vaccine protection have gone beyond the traditional serologic analysis and employed transcriptomic sequencing techniques and bioinformatic analyses to better characterize and understand the molecular and genetic underpinnings of the vaccine response. A seminal work from Querec *et al*. assessed the transcriptome of CD8+ T and B cells from humans vaccinated with the Yellow Fever vaccine YF-17D by microarray and identified a gene expression pattern correlating to seroconversion [20]. Efforts were made to analyze the immune response to influenza vaccination that identified not only high resolution molecular signatures at 7-days post inoculation, but also identified gene signatures to predict antibody response [21]. A meticulous serologic, cellular and bioinformatic analysis of pre–and post–vaccination samples from influenza vaccinates identified correlative biomarkers for vaccine response quality in pre–vaccination samples [22]. Strides have been made in understanding vaccine response in the veterinary sciences using transcriptome sequencing techniques also. In response to vaccination against *Mycobacterium bovis*, cattle protection was highly correlated with IFN-gamma and IL-22 expression [23].

To further our understanding of the genetic and cellular mechanisms underlying vaccination and seroconversion in arMP-12 vaccinated livestock, we hypothesized that bioinformatic analysis of the RNASeq sequenced transcriptome of peripheral blood mononuclear leukocytes (PMBC) will identify highly correlated gene transcripts for protective seroconversion as measured by PRNT_80_. To test this hypothesis, we analyzed the global transcriptome of PBMCs from cattle vaccinated with arMP-12 against RVFV before and after vaccination, followed by a bioinformatic correlative analysis between perturbed cellular pathways and serologic analysis for protective immunity. To more completely utilize the data, we analyzed resulting RNASeq sequences by: 1) standard day-by-day analysis oriented from the day of vaccination, or 2) a time-shifted mechanism in which the time points were oriented around the day that each calf had protective 1:80 serum neutralizing antibody titers. For both of these orientations, sequence data from each time point were analyzed by standard differential gene expression analyses [S1 Text] and Dynamic Bayesian Gene Group Activation (DBGGA) methodology to identify both perturbed cellular pathways and gene ontology (GO) terms [24]. Using the time-shifted data, we then employed a two-step approach to build a Dynamic Bayesian Network to predictively correlate gene/pathway perturbation patterns with immune response and 1:80 protective antibody titers. We identified specific highly correlated cellular pathways and constitutive genes that were perturbed preceding protective seroconversion. These results contribute to a deeper understanding of the underlying biology of protective vaccine immune responses, as well as underscoring the utility of bioinformatic analysis of high-density sequence data to identify transcriptomic precursors to protective seroconversion.

## Materials and Methods

### Vaccine Strain and Plaque Reducing Neutralizing Titer Assay

Retrospective RNASeq analysis was performed on PBMCs taken from five healthy 4–6 month *Bos taurus* steer calves as described previously [15]. The calves were injected intramuscularly or subcutaneously with 1x10^5^ PFUs of authentic recombinant MP-12 (arMP12) virus in 1.0 ml of phosphate buffered saline (Sigma) [15, 25]. The arMP-12 virus is genetically identical to the live, attenuated RVF MP-12 vaccine, prepared by the Salk Institute, Swiftwater, PA, for the U.S. Army Medical Research Institute of Infectious Diseases (USAMRIID) for use in humans under an Investigational New Drug (IND) Application [17, 25]. Serum was collected to assess the presence of serum neutralizing antibodies using a plaque reduction neutralization test (PRNT_80_) as previously described [26]. PRNT_80_ values used in this work were calculated and reported to assess the immunogenicity of the arMP-12 vaccine to produce neutralizing antibodies in cattle [15].

### RNA Isolation, Preparation and Sequencing

Whole blood was collected from vaccinated cattle at DAY 0–7, 10, 14 and 21 and immediately mixed at a 3:5 (blood:buffer) ratio with RNALater (Invitrogen). Samples were stored at –20°C until processing. To purify RNA, frozen samples were thawed at 37°C, centrifuged at 2000xG, and supernatant discarded. Remaining cell pellets were subjected to two rounds of treatment with Red Blood Cell Lysing Buffer per manufacturer’s instructions (Sigma). RNA from resulting cell pellet was initially extracted by Trizol (Invitrogen) followed by treatment with RNeasy Kit with on-column DNase digestion (Qiagen). Purified RNA was quantified by spectrophotometry on a NanoDrop (Thermo, USA) and by electrophoresis with a BioAnalyzer 2100 (Agilent, USA). Only samples with an RNA Integrity Number (RIN) greater than 8.0 were used for RNASeq analysis.

All RNASeq sequencing was performed at the Texas A&M Genetics and Bioinformatics Center following manufacturer’s instructions. cDNA was generated from RNA samples of sufficient quantity and quality with Ovation RNA-Seq System (NuGen, USA), sheared by focused ultrasound (Covartis, USA) and made into barcoded (multiplexed) libraries with Encore Rapid Library System (NuGen, USA). Samples were quantified, diluted, pooled and re-analyzed with a Bioanalyzer and loaded onto a Genome Analyzer II High-throughput sequencer (Illumina, USA). Sequence data (fastq format) were transferred to Seralogix LLC (Austin, TX, USA) for bioinformatic analysis. All of the original data are available in the Gene Expression Omnibus at the National Center for Biotechnology Information (http://www.ncbi.nlm.nih.gov/geo/), Accession #GSE71417.

### Sequence assembly and locus gene annotation

Raw read post processing steps were performed using a computational pipeline (Seralogix, LLC, Austin, TX) described in more detail in S1 Text. All fastq read files were checked for quality (base quality scores, adaptor contamination, duplicate sequences) using FastQC [27]. Filtering of read data to achieve the best quality fastq files was performed using Trimmomatic [28] tools to remove adaptor contamination, low quality leading and trailing reads, and to crop read length to 73-bp determined to be the optimal length of high quality reads. The resulting post-processed fastq read files were aligned to the bovine reference genome UMD3.1 [29] using Bowtie2 [30]. Utilizing the BedTools Multicov function [31], the read alignment overlap counts were mapped to reference sequence file in BED [32] genomic interval format. The bovine genomic intervals were obtained from Ilumina’s Igenome [33] as ready-to-use reference sequences and gene annotations. Multicov converts these mapped read counts to genomic regions that define known genes and then outputs a gene reads count table file inclusive of all samples over the complete time course.

### Transcriptomic statistical differential expression analysis

The gene reads count table was imported into a database for further processing in a computational pipeline where DESeq [34] was employed to normalize the data and determine the time-course differential gene expression between vaccinated and non-vaccinated calves. Only gene loci with an expression level greater or equal to 7 counts (≈0.3 counts per million reads) across at least one half of all 47 samples were included in the normalization and differential expression analysis. At each locus, p-value and z-score statistical tests and fold change were computed to determine significance of differential expression. To account for multiple hypothesis tests, a false discovery rate (FDR) was calculated using the fdrtool, an R program [35]. Only differential expressions with a q < 0.05 were considered statistically significant.

### Pathway and gene ontology analysis using Bayesian models

To identify differentially expressed genes, GO terms [36], or pathways [37, 38], we employed the Dynamic Bayesian Gene Group Activation (DBGGA) method (Seralogix, LLC, Austin, TX) that includes all observed genes within a group to determine the overall perturbation impact of the gene set in comparison to baseline controls. DBGGA analysis was performed to determine temporal signatures of canonical pathways (PW) and gene ontology (GO) groups in response to the RFV vaccine post inoculation (p.i.) or pre-serum neutralization (pSN). DBGGA enables the determination of which PW and GO groups are most perturbed (activated or repressed) in one condition relative to another and which genes are the significant sources of the perturbation (designated as “influential regulator genes” (IRG). The DBGGA scoring method relies on posterior Bayesian network sampling and interrogation methods to measure a complete PW or GO group perturbation and can determine a single gene’s influence in a gene set and transform this influence measure (log-likelihood deviation) to a z-score test statistic (hereafter referred to as the Bayesian z-score to depict its association with DBGGA scoring method). Unlike other pathway analysis or gene set enrichment [39] methods based on classical statistical tools, this method identifies gene regulatory relationships that are most involved in the context of biologically related genes and their interactions (the DBGGA influence score is not reliant on identifying differentially expressed genes prior to PW/GO analysis). Additional information on the DBGGA method has been described previously [24, 40–44] and reviewed briefly in S1 Text, Sections 1.0 and 2.0. Additionally, an alternative approach to pathway and GO analysis was conducted employing the R package “GAGE” [45] for comparative purposes.

### Pathway temporal signature correlation with the antibody response

We developed a sliding window correlation (SWC) procedure (written in MATLAB [46]) to identify early pathway response signatures that correlate with the time course of the plaque-reduction neutralization test (PRNT_80_) to later time points at the onset of protective immunity. The SWC procedure measures the temporal trajectory correlation of a pathway or GO term Bayesian z-scores and their associated gene Bayesian z-scores for an incremental series of three time points to a fixed PRNT_80_ temporal response signature that is also comprised of three time points surrounding the PRNT_80_ serum neutralization point. We elected to use three time points as our sliding window to better capture the early temporal dynamics of PW/GO events that may have direct correlation to the later serum neutralization temporal signature. Correlation coefficients (R) were computed for sets of overlapping successive pathway, GO and their gene scores across all time points represented by the sliding time window sets (time -6, -5, -4; times -5, -4, -3; times -4, -3, -2;times -3, -2, -1; times -2, -1, 0; and times -1, 0, 1) with the fixed PRNT_80_ temporal response at times -1, 0, 1 pre-serum neutralization that represents the onset of protective immunity. Additional details of the sliding window correlation procedure and definition of pre-serum neutralization time points are described in S1 Text, Section 3.0.

### Learning transcriptomic temporal signatures to predict protective immune response

Ideally, we sought to identify early patterns in the pathway and transcriptomic host responses that can be used to predict protective immunity. We developed a dynamic Bayesian network (DBN) modeling approach [47] to use as a predictive model of the PRNT_80_ protective immune response from the highly correlated gene expression found present in a selected set of corresponding SWC correlated pathways and GO terms. This is a supervised learning method that can uniquely deal with time-course data. The method is described in greater detail in S1 Text, Section 4.0

### Ethics Statement

Healthy, 4–6 month old *Bos taurus* heifer and steer calves were used in the present study as described previously [15]. The Texas A&M University Institutional Animal Care and Use Committee approved the Animal Use Protocol 2010–192 in accordance with the U.S. Animal Welfare Act as enforced by the United States Department of Agriculture, Animal and Plant Health Inspection Service.

## Results and Discussion

### Read mapping and transcript gene annotation

RNA from whole blood was isolated from cattle [15] prior to vaccination (D0) as well as after inoculation (D1-7, 10, 14, and 21) and analyzed by RNASeq. After demultiplexing of the sequencing data, we obtained a range of total read counts across the 47 biological samples varying from 36 to 67 million single-end reads. After further processing for filtering and alignment, we obtained a range across the samples from 31 to 53 million reads representing 76–89% of the starting reads aligning to the reference bovine genome [48] indicating a high proportion of mapped reads and a reasonable uniformity between samples (S1 Table). We then performed a mapping of reads to known genomic gene locations using the Igenome NCBI genomic interval reference and the associated NCBI annotation file [33]. We obtained 16,787 genes, after filtering for expression level threshold of at least 7 counts per loci across a minimum of 50% of the individual samples. Only genes meeting these criteria were used for further analysis.

### Standard-time differential expression resulting from vaccination

**Fig 1:**
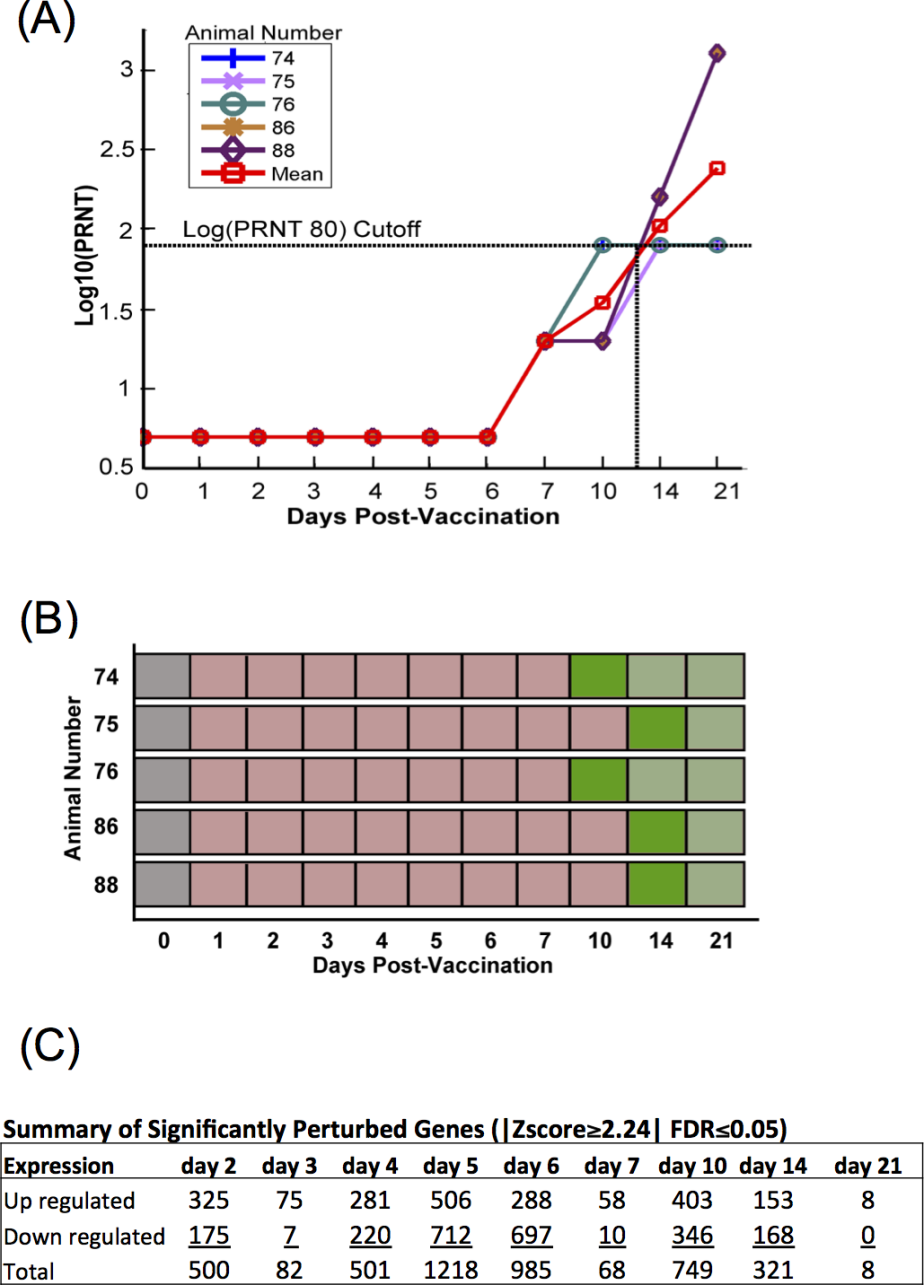
Serologic and perturbed gene analysis of cattle vaccinated with arMP-12. (**A**) Log10 plaque reducing neutralizing titers (Log10(PRNT_80_)) of test animals prior to and after vaccination with arMP-12. A dilution of 1:80 (log10 = 1.903) was used as the cutoff to determine protection from WT RVFV challenge based on prior published work. (**B**) Schematic of the immunologic status of test animals by day of experiment: unvaccinated (gray), vaccinated, below PRNT_80_ cutoff (light red), vaccinated, first day at or above PRNT_80_ cutoff (dark green), vaccinated, above PRNT_80_ (light green). (**C**) Summary count of significantly perturbed genes normalized against unvaccinated samples occurring in at least 50% of animals by day post inoculation, with a |Z-score ≥2.24| and a false discovery rate (FDR) ≤0.05.

During the time course collection of our samples, we sampled most frequently on days immediately following vaccination, as well as regular intervals out to 3 weeks post vaccination (D1–7, 10, 14, and 21). This repeated sampling early in the experiment allowed for a more detailed analysis of the gene expression profiles present just after vaccination, in contrast to analysis of the transcriptome from a single time point post-inoculation, and provided for improved orientation of transcriptome data to previously reported serologic data [15]. Plaque reducing serum neutralizing titers (PRNT_80_) serve as an accurate indicator for protection against wild-type RVFV and are commonly used in determining RVFV vaccine effectiveness [15, 16, 49–52]. S/N analysis of five calves used for transcriptomic study identified an average protective titer of 1:80 by D11 p.i. (Fig 1A and 1B).

Differential gene expression was determined in conjunction with DESeq and resulted in a time-course profile of significantly perturbed up and down regulated genes (Fig 1C). Differential expression levels in vaccinated animals were determined by comparing transcript levels to their own baselines over the 21 day time-course (FDR<5%; |Z-score| ≥2.24). Temporal change in the number of significant expressions is more pronounced in the days prior to seroconversion which occurred at between 10 and 21 days post inoculation. The top 15 up and down regulated expressions by time are listed in S2 Table with a more complete gene description list provided in S3 Table.

### Dynamic Bayesian Gene Group Activation (DBGGA) Pathway Analysis

**Fig 2:**
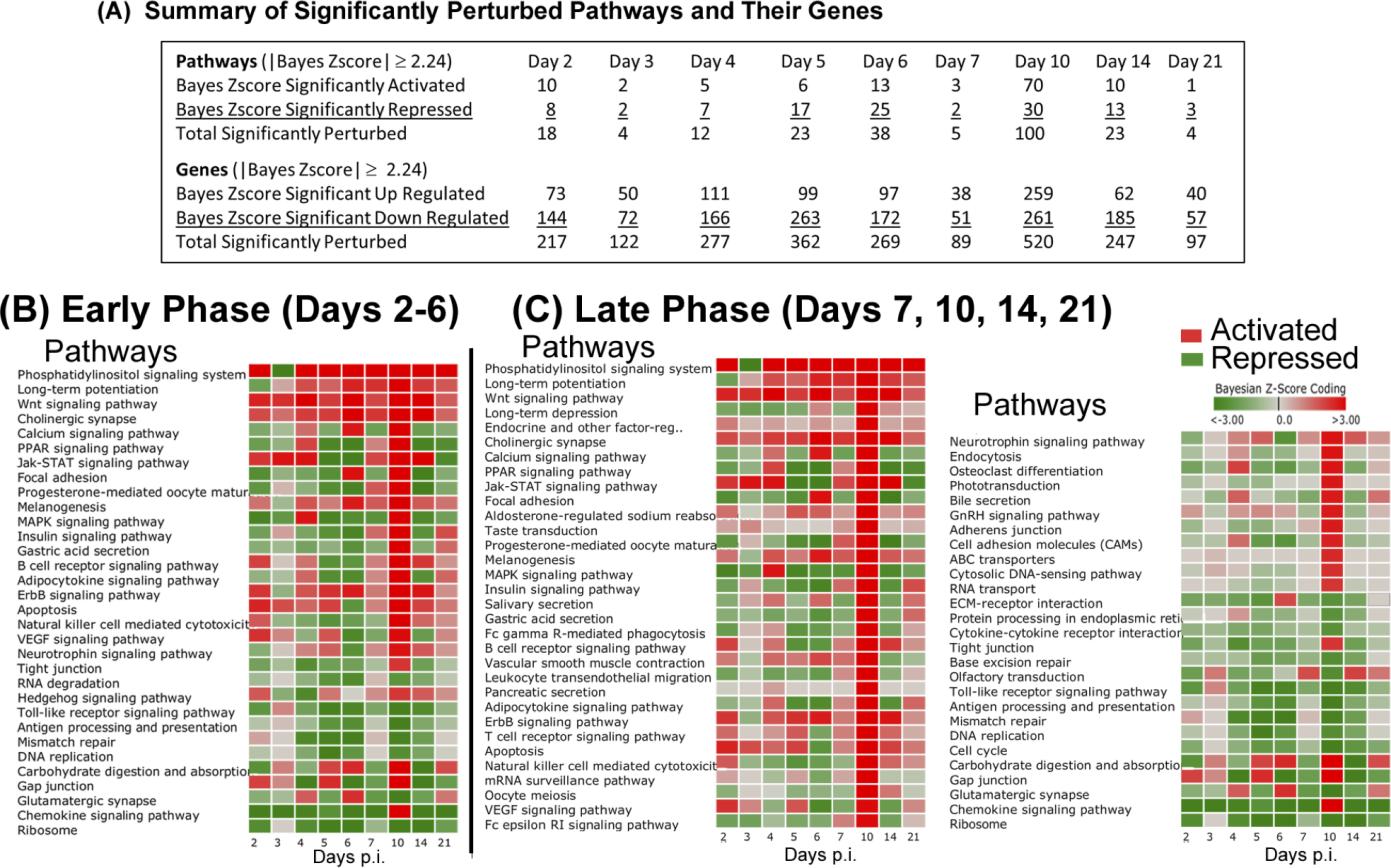
Dynamic Bayesian Gene Group Activation (DBGGA) Pathway analysis. (**A**) Summary table of cellular pathways significantly activated or repressed (Bayesian score ≥|2.24|) over the time course of the experiment as determined by the DBGGA tool. (**B-C**) Read out and heat map of significantly perturbed pathways during days post inoculation (p.i.). Red color indicates activation, green color indicated repression. Intensity of color represents amplitude of perturbation. Analyses are oriented to the Early Phase (days 2–6 p.i.) (**B**) or the Late Phase (days 7, 10, 14, 21 p.i.) (**C**) to identify significant perturbations at different periods during the vaccine response.

Employing the DBGGA process, 229 pathways and 4127 unique genes were scored in this KEGG pathway set. The term pathway is used broadly as defined by KEGG and may include other gene sets that may define, for example, protein interactions or disease networks that are not strictly considered as signaling or metabolic pathway network types. The summary results of activated and repressed pathways and the up-regulated and down-regulated genes meeting a |Bayesian z-score| > 2.24 are shown in Fig 2A (full list of DBGGA Pathways in S4 Table; full list of genes from identified in DGBBA Pathways in S5 Table). Activation or repression is determined by the overall numeric average of the DBGGA Bayesian z-score for all associated genes within a pathway. If the average is positive, indicating the dominance of up-regulated genes, the overall pathway score is designated as activated, else if the average is negative the pathway is designated as repressed and the pathway score is annotated accordingly with a plus or minus sign. Since our time course covers immunologically distinct periods, we expect distinct pathways and genes to be perturbed at different points after vaccination. To better capture these variations, we grouped the time points into “Early Phase” (D2–6) and “Late Phase” (D7, 10, 14, and 21). In the Early Phase, there were 10 activated pathways, with the most notable being *phosphatidylinositol signaling system*, *apoptosis*, *Wnt signaling pathway*, *VEGF signaling pathway*, *Jak-STAT signaling pathway* and *ErbB signaling pathway* (Fig 2B). The most repressed pathways included *chemokine signaling*, *MAPK signaling*, *ribosome*, *focal adhesion*, and *toll-like receptor*. Interestingly, the *ribosome* pathway was significantly down-regulated throughout the course of the study, an expression pattern that was previously seen in cattle vaccinated against Bovine Viral Diarrhea Virus and hypothesized to be apart of a coordinated host response for down-regulation of protein synthesis during viral infection (Fig 2B) [53]. While the specific contribution of genes comprising the *ribosome* pathway to immune response to arMP-12 is unclear, this characteristic may be an important global gene regulation feature for immune response against viral infection.

At Day 7 in the Late Phase, there were only 5 significantly perturbed pathways meeting a |Bayesian z-score| > 2.24, which is in high contrast to that of day 10 where 100 were perturbed. We also observed that day 10 had the highest number of differential expressed genes. Since the DBGGA pathway scoring method is very sensitive to changes in the gene expression in the experimental condition, the higher perturbation in the number of genes reflects this larger number of pathway perturbations. This increased perturbation at day 10 was mainly attributed to increases in metabolic, organismal systems, and human disease pathways, which may not be directly related to the vaccine immune response.

While the pathways perturbations from the Early and Late Phases provided insight into the molecular and genetic underpinnings of immune response to vaccination, the authors note that it was beyond the design of this retrospective study to deduce specific causative effects between the arMP-12 vaccination, the immunologic response as observed by RNASeq, and generation of neutralizing antibodies. Several perturbed pathways noted in Fig 2 have no obvious specific link to immune response, such as *Neurotrophin signaling pathway* or *Gap junction*. We speculate that these pathways share constitutive perturbed genes with pathways more directly related to immune response. While there may be a tangential or parallel activation of these pathways as a result of vaccination or generic immune response, we have not fully investigated this possibility in this work.

### DBGGA gene ontology (GO) term analysis

**Fig 3:**
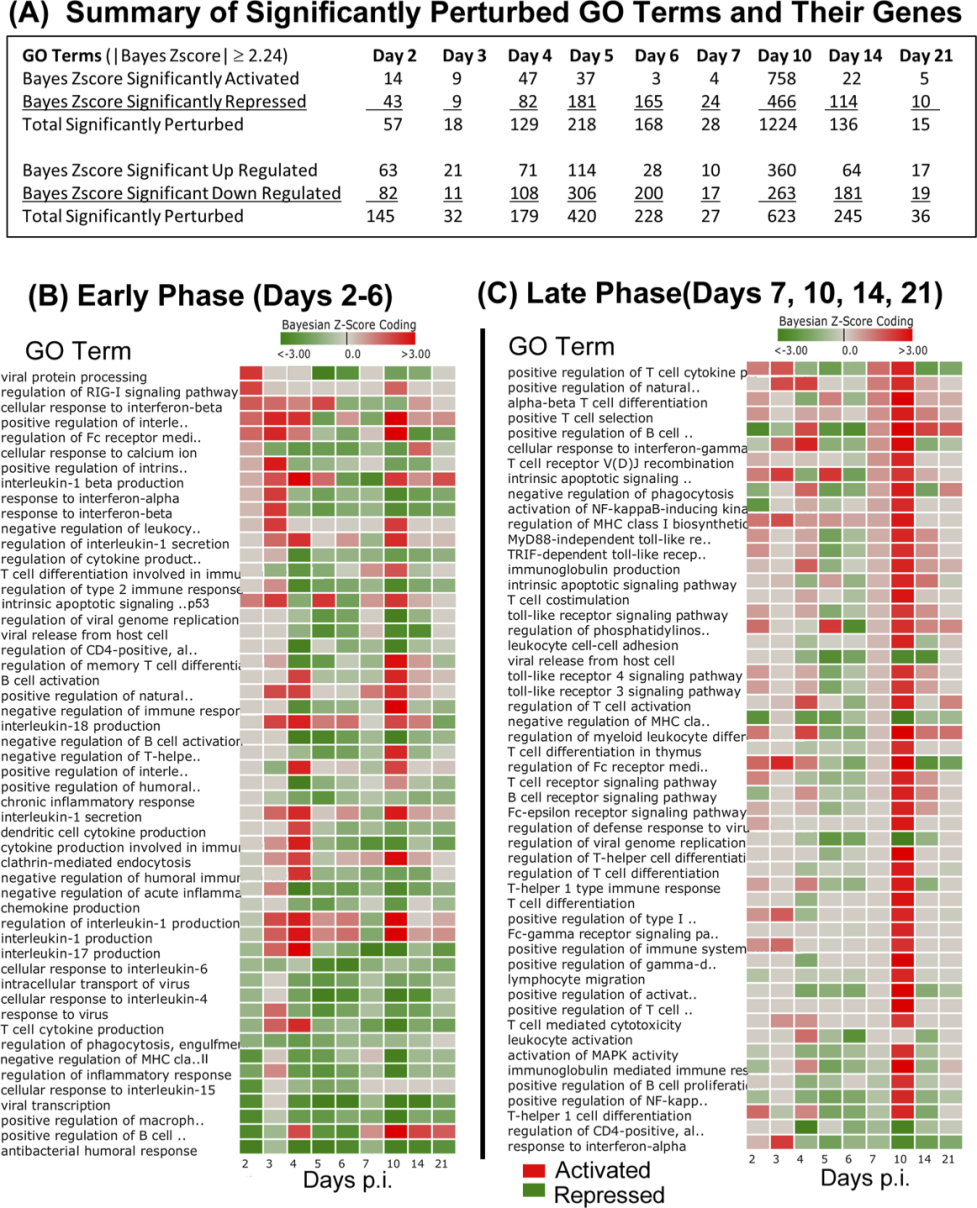
Dynamic Bayesian Gene Group Activation (DBGGA) Gene Ontology (GO) Term analysis. (**A**) Summary table of significantly perturbed GO terms and genes described by day post inoculation. Only GO terms and their genes with Bayesian score ≥|2.24| are included in analysis. (**B-C**) Heat maps of perturbed GO terms described by day post inoculation (p.i.) and identified from the Early Phase (**B**) and the Later Phase (**C**). Red color indicates activation, green color indicates repression. Intensity of color represents amplitude of perturbation. The list of GO terms shown represents a subset of all perturbed terms that were selected as being most relevant to innate and adaptive immune response.

The DBGGA process scored 4354 biological process GO Terms (restricted to GO terms having between 5–300 observed genes within a given term). Within this set of GO Terms, there were 6818 uniquely scored genes. The summary results of activated and repressed GO Terms and their associated gene sets are shown in Fig 3A. A complete list of perturbed GO Terms and their associated DBGGA gene scores are provided in S6 and S7 Tables, respectively. In the Early Phase, a set of relevant activated and repressed GO Terms were selected to show the complex dynamics of the early host innate immune response to arMP-12 vaccine (Fig 3B). There were GO Term perturbations associated with *regulation of RIG-I signaling pathway*, *positive regulation of interferon-alpha*, *cellular response to interferon-gamma*, *response to interferon–alpha and–beta*, *viral protein processing*, *viral transcription*, and *viral life cycle*. *RIG-I signaling activation* is likely caused by the detection of pathogen-associated molecular patters (PAMP) within the vaccine viral RNA and initiates the innate antiviral immune response, an essential precursor for launching an effective adaptive immune response. *Viral life cycle* and *viral transcription* are highly repressed GO terms with 72 genes being significantly down-regulated in the early phase. The majority of these genes encode for ribosomal proteins. The viral process GO terms are associated with processes by which a viral gene is converted into a mature gene product or products (proteins or RNA). This includes viral transcription, processing to produce a mature RNA product, and viral translation indicating viral replication. The dominant genes involved in these processes included 54 genes encoding a family of ribosomal proteins. These important GO terms and associated genes are listed in S6 and S7 Tables respectively.

Also provided in S6 Table and show in Fig 3B are details of other important early phase highly repressed GO terms that include *antibacterial humoral response*, *positive regulation of B cell receptor signaling*, *positive regulation of macrophage cytokine production*, and *negative regulation of MHC class II biosynthetic processes*. Interestingly, of these repressed GO terms only the *positive regulation of B cell receptor signaling* becomes significantly activated in the later phase (defined to include days 7, 10, 14, and 21). Also in the early phase, we observed the strong activation of such biological processes as the *interleukin-1beta*, *17*, and *18 production*, *regulation of Fc receptor mediated stimulatory signaling*, *intrinsic apoptotic signaling by p53 class mediator*, *negative regulation of leukocyte mediated cytotoxicity*, *regulation of cytokine production*, *clathrin-mediated endocytosis*,[54] and *positive regulation of natural killer cell differentiation*.

In the Late Phase, we began to detect more biological processes associated with the adaptive immune response which included *T cell activation*, *differentiation*, and *receptor signaling*, *regulation of T-helper cell differentiation*, *B cell activation*, *regulation and receptor signaling*, and *regulation of memory T cell differentiation*, *T-cell differentiation*, *T-helper 1 type immune response*, *immunoglobulin mediated immune response*, and *regulation of T cell cytokine production* (Fig 3C). By Day 21, the host response returned to a basal state, as seen in the reduction in number of perturbed GO Terms and associated genes (Fig 3A). As before, we note that this retrospective analysis is unable to differentiate between a specific response to arMP-12 vaccination and a generic immunological response. We expect many of the perturbed genes pathways identified in this analysis would be found in either an infectious challenge with wild-type Rift Valley fever Virus or possibly other pathogenic viral insults due to the current understanding of these pathways in viral immune response. Despite this, we propose that these data provide an important baseline of temporal immune response that will aid in future vaccine development, therapeutics, and diagnostics.

### Defined time-shift analysis of differential gene expression data

With out-bred animal subjects, variation in the immune response to vaccination is expected. In this experiment, the time for PRNT_80_ to reach the threshold of 1:80 PRNT_80_ (log10 = 1.903 indicated by horizontal dotted line) for calves #74 and 76 was D10 after inoculation (Fig 1B). However, in this animal cohort, calves #75, 86, and 88 reached their 1:80 threshold at D14 (Fig 1B). While this difference has minimal impact on determining the efficacy of the vaccine, the delay may effectively mask the true genetic and mechanistic response preceding the production of neutralizing antibodies. A day-by-day analysis of an asynchronous genetic response could fail to accurately identify perturbed pathways or genes, or obscure important expression trends and perturbations.

**Fig 4:**
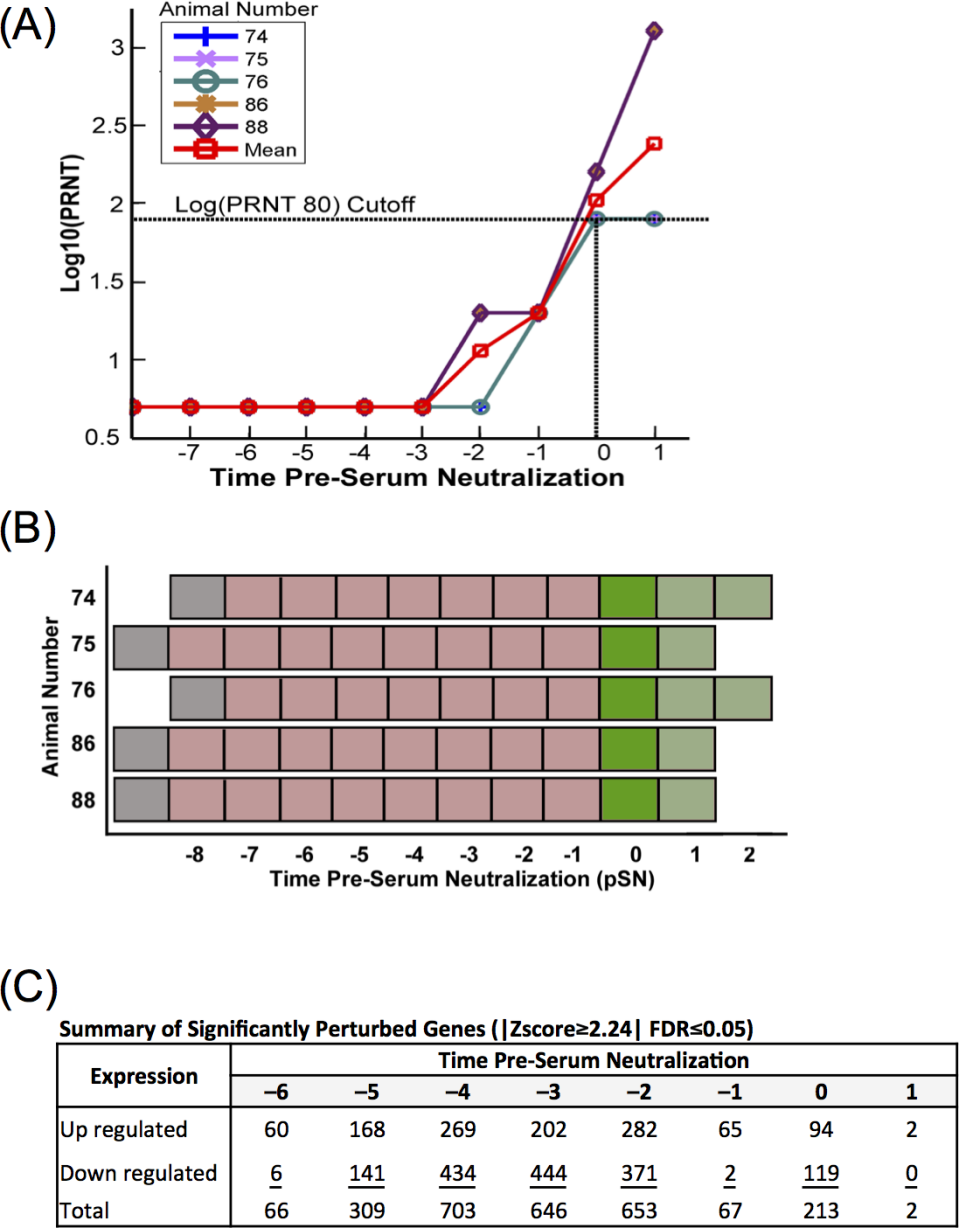
Serologic and perturbed gene expression in time-shifted data. (**A**) Log10 plaque reducing neutralizing titers (Log10(PRNT_80_)) of test calves prior to and after vaccination with arMP-12 with data synchronized to the time point that each animal reached the minimum threshold (T = 0; 1:80, log10 = 1.903). (**B**) Schematic illustrating the immunologic status of test animals in time shifted analysis: unvaccinated (gray), vaccinated, below PRNT_80_ cutoff (light red), vaccinated, first day at or above PRNT_80_ cutoff (dark green), vaccinated, above PRNT_80_ (light green). (**C**) Count of significantly perturbed genes normalized against unvaccinated samples occurring in at least 50% of animals by time pre-serum neutralization, with a |Z-score ≥2.24| and a false discovery rate (FDR) ≤0.05.

To account for this, we performed a defined time-shift analysis by synchronizing the sequence data from each animal to reflect the antibody response (Fig 4A and 4B). We shifted the sequence data to align all animals by the time point when the serum neutralization titers of each animal reached clinically protective levels at a 1:80 PRNT_80_ titer [15, 50, 55]. We achieved alignment by shifting calves # 75, 78, and 88 to align their time of serum neutralization with calves # 74 and 76. Data in this time-shifted orientation are reported as time prior to serum neutralization (pSN), with the time point of seroconversion labeled as Time 0 and relative perturbation determined by comparison to pre-vaccinated samples.

As with the standard time data, differential gene expression was determined by bioinformatic methods and DESeq. A time course analysis of perturbed genes (FDR<5%, |Z-score| ≥2.24) showed a steady increase of both up and down regulated genes from –6 pSN until –2 pSN, after which there was a rapid drop in number of perturbed genes for the remaining time points (Fig 4C). The top 15 up or down regulated genes are listed in S8 Table, and a complete perturbed gene list is provided in S9 Table. Several up and down regulated genes are common to the Early or Late Phases from both the standard-time and time-shifted data sets, including tumor necrosis factor super family 10 (TNFSF10), gamma-interferon-inducible protein Ifi-16 (IFI16), the gene coding for B-cell linker protein (BLNK), and lymphocyte-specific protein 1 (LSP1). In both time treatments of the data, numerous ribosome family proteins were among the most down-regulated genes in the Early Phase (see S10 Table for comparison).

### Defined Time-shifted DBGGA pathway analysis

**Fig 5:**
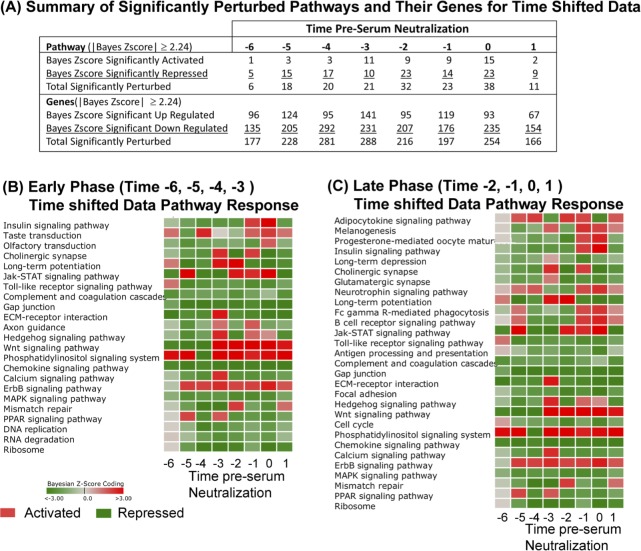
DBGGA pathway analysis on time-shifted data. (**A**) Summary table of significantly activated or repressed cellular pathways (Bayesian score ≥|2.24|) on time shifted data. Perturbation of cellular pathways and constituent genes are reported. (**B-C**) Heat map representation of significantly perturbed pathways at times pre-serum neutralization (pSN). Red color indicates activation, green color indicates repression. Intensity of color represents amplitude of perturbation. Analyses are oriented to the Early Phase (time –6, –5, –4, –3 pSN) (**B**) or the Late Phase (time –2, –1, 0, 1 pSN) (**C**) to identify significant pathway perturbations at different periods during the vaccine response.

The summary results of activated and repressed pathways and the up-regulated and down-regulated genes meeting a |Bayesian z-score| > 2.24 are shown in Fig 5A (full list of pathways in S11 Table, list of genes in S12 Table). Since these time-shifted data use the same original data as the standard time analysis, the same number of signaling/metabolic pathways (229) and unique genes (4127) were observed here as before. We have again segregated the data into Early Phase (time –6, –5, –4, and –3, pSN) and Late Phase (–2, –1, and 0 pSN) to better appreciate the genetic components active at each phase of the immune response.

The majority of significantly modulated pathways was repressed, especially at in the Early Phase (–6, –5, and –4 pSN). At very early and late time points, *Phosphatidylinositol signaling* was significantly perturbed (Fig 5B and 5C). Several viruses have been shown to modulate the Phosphatidylinositol signaling system [56–58]. Recent findings suggest that in response to double-stranded RNA (dsRNA), the phosphatidylinositol-3-kinase (PI3K) is activated and mediates activation of the transcription factor interferon regulatory factor 3 (IRF3) [56]. Gene level analyses of this pathway indicated that the most prevalent up-regulated PI3K related genes across most time points were PIK3R1, PIK3R2, PIK3R3, PIK3C2B, and PIK3CA, but the gene, IRF3, was only notably up regulated in the TLR pathway at times –6 and –1 pSN (S9 Table). Phosphatidylinositol signaling has also been implicated in the activation of *Calcium signaling* and more specifically the Ca2+/calmondulin kinase. PI3Ks are known to regulate diverse signaling pathways involved in growth, proliferation, survival, differentiation and metabolism. In T cells, PI3Ks can be activated by a number of different receptors including the T cell receptor (TcR), co-stimulatory receptors, cytokine receptors, and chemokine receptors.

In comparing the standard time and time-shifted DBGGA pathway analyses, several pathways were detected by both treatments. In the Early Phase, *Phosphatidylinositol signaling system*, *Jak-STAT signaling* and *ErbB signaling* pathways were identified as up-regulated by both analyses (Fig 2 vs. Fig 5; S4 and S5 Tables vs. S11 and S12 Tables). Within the Late Phase, the standard analysis identified numerous highly perturbed, up-regulated pathways, while the time-shifted analysis detected just 13 up-regulated pathways. We found this to be an important difference between the treatments of the time course data, as the time-shifted analysis decreased the number of perturbed pathways, allowing for a more focused list of high priority pathways for further examination. In this case, the time-shifted analysis identified immunologically relevant pathways *Fc gamma R-mediated phagocytosis*, *B cell receptor signaling pathway*, *Phosphatidylinositol signaling* system, and *Wnt* and *ErbB signaling pathways*.

### Defined time-shifted DBGGA gene ontology (GO) term analysis

**Fig 6:**
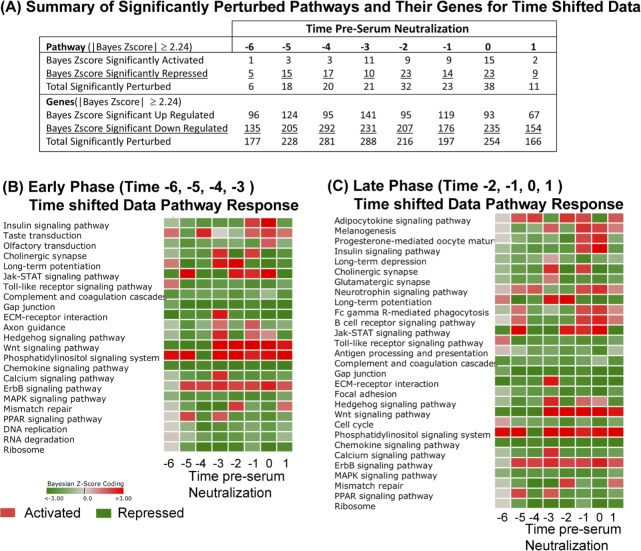
DBGGA GO term analysis on time-shifted data. (**A**) Summary table of GO Terms and component gene perturbation by time pre-seroconversion. Only GO terms and their genes with Bayesian score ≥|2.24| are included in analysis. (**B-C**) Heat maps of perturbed GO terms described by time pre-seroconversion and identified from the Early Phase (time –6, –5, –4, –3 pSN) (**B**) and the Later Phase (time –2, –1, 0, 1 pSN) (**C**). Red color indicates activation, green color indicates repression. Intensity of color represents amplitude of perturbation. The list of GO terms shown represent a subset of all perturbed terms that were selected as being most relevant to innate and adaptive immune response.

As before, the DBGGA process scored 4354 biological process GO terms (restricted to GO terms having between 5–300 observed gene within the term). Within this set of GO terms, there were 6818 uniquely scored genes. The summary results of activated and repressed GO term gene sets only for GO terms of root “biological_process” are provided in Fig 6A. A set of noteworthy activated and repressed GO terms were selected (Fig 6B, S13 Table), to show the dynamics of the host response to MP-12 vaccine. There was a considerable change over the time course between days indicating a very diverse response. At time– 6 pSN, two highly activated DBGGA GO *terms were response to interferon-alpha* (GO:0035455), *response to interferon-beta* (GO:0035456), *negative regulation of viral genome replication* (GO:0045071), *positive regulation of T cell cytokine production* (GO:0002726), and *positive regulation of interleukin-1 beta production* (GO:0032731), reversing and becoming repressed in later time points.

This emphasis on pathways associated with the Type-I interferons may serve as an important factor in distinguishing vaccinated and infected animals. During a vaccine trial with mutagenized or gene deletion strains of the virus on mice lacking interferon (IFN)–alpha/beta, or–gamma receptors, vaccine strains elicited strong IFN–alpha and–beta responses while the WT control strain failed to elicit the same response [59]. Follow-up work with a deletion RVFV strain showed the non-structural protein NSs was responsible for blocking expression of IFN by targeting a basal cellular transcription factor [59–61]. The strong IFN expression identified in this study with the MP-12 vaccine suggests the known V160A mutation in the NSs, or a combination of mutations in MP-12 including NSs, is sufficient to compromise the normal repressive functions of the protein in cattle [14].

In comparing the DBGGA GO term analysis between standard time and time-shifted data, there is a typical down regulation of GO Terms and genes, with the most perturbation at D10 for the standard time (Fig 3A vs Fig 6A). With the time-shifted data, the general trend of down regulation at each time point remains consistent with the standard time, however the number of perturbed GO terms is generally lower and peaks at time –3 pSN (Fig 6A).

In the Early Phase, the standard time data identified numerous perturbed pathways associated with immune response, including *viral transcription*, several pathways involved with *interferon alpha* or *beta production*, and *interleukin-1 production* and response. As before, this analysis resulted in a large cohort of perturbed pathways. Under the time-shifted protocol, the overall number of perturbed pathways was decreased in both the Early and Late Phases, but immunologically relevant pathways were still detected. Of note was the down regulation of *viral transcription*, *protein processing*, and *life cycle* pathways and up regulation of pathways associated with interleukin-1 and *response to interferon alpha*.

### Pathway and GO term temporal signature that correlate with the PRNT_80_ response

**Fig 7:**
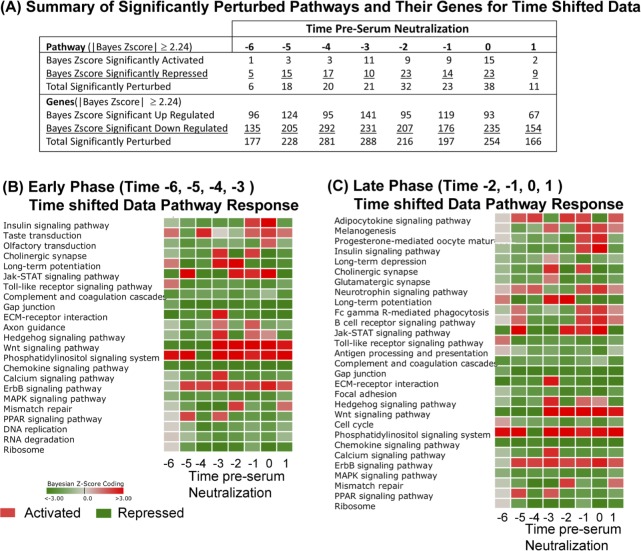
Sliding window correlation (SWC) to identify pathways associated with serum neutralization titers. (**A**) Visualization of sliding window correlation approach. (**i**) Hypothetical Log10(PRNT_80_) data taken from time points capturing neutralizing antibody levels during time period in which animals reached threshold for protection (log10 = 1.903) (dark green bars). (**i-ii**)The trajectory of the Log10(PRNT_80_) data is applied to other time points prior to serum neutralization (light green bars) to identify pathway with complementary or antithetical trajectories (**A-ii** for hypothetical Pathway Bayesian Z-score data, dark blue bars). (**iii**) Graphical representation of the R correlation coefficient value between Log10(PRNT_80_) and Pathway Bayesian Z-score. (**B**) Pathways correlated to PRNT_80_ values at the time of seroconversion listed by pathway at incremented window times (each consisting of three time points), with significant correlation p-values (**i**), or plotted as log(PRNT_80_) vs normalized Bayesian Z-score (**ii**) for six selected pathways having highest correlations.

To gain insight into the potential mechanisms underlying the host response to the arMP-12 vaccine and to elicit early biomarkers of protective immunity, we developed a sliding window correlation (SWC) approach to apply to pathways, GO terms, and genes (Fig 7, S15 Table). We choose the log(PRNT_80_) time points of days –1, 0, and 1 pSN to represent the onset and establishment of protective immunity (see Figs 4A and 7A-i). This trajectory of data over the three time points was correlated against the pathway, GO term, and gene DBGGA scores obtained for each host across all time points post immunization.

The SWC analysis included only the canonical pathways with the exclusion of metabolic pathways. Fig 7B-i lists the pathways by time window for those found to meet a significance-of-correlation |p-value| < 0.05 and with pathway scores at any one time point exceeding the |Bayesian z-score| > 2.24. The p-values are given a sign to indicated positive (+) or negative (-) correlation direction. Note also that time windows (Times –6, –5, –4 pSN and –5, –4, –3 pSN) are all negatively correlated while for the time windows (Times –3, –2, –1 pSN) and beyond are all positively correlated.

**Fig 8:**
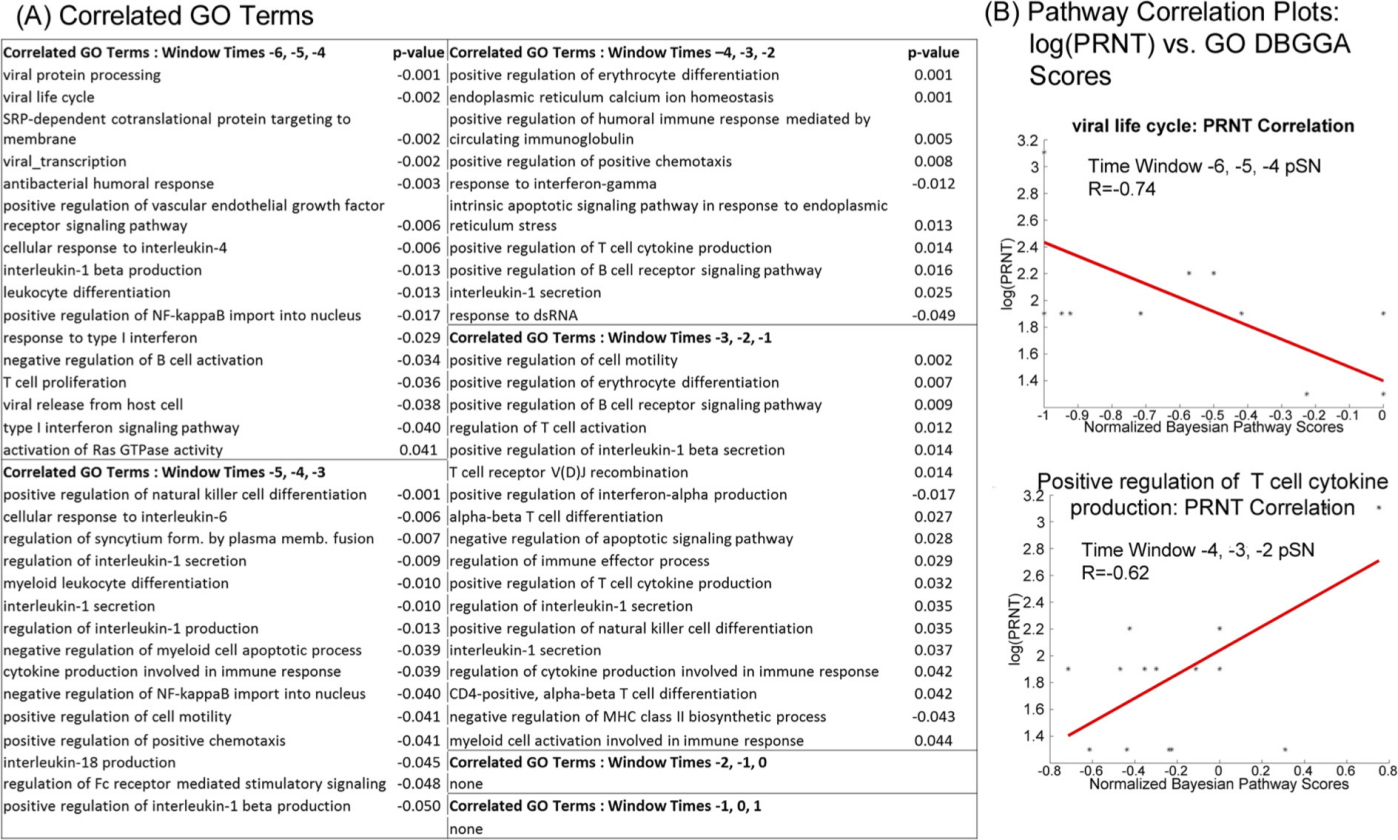
Sliding window correlation (SWC) to identify GO terms associated with serum neutralization titers. (**A**) Immunologically important GO terms correlated with log10(PRNT_80_) listed by time window with associated p-value indicated significance of correlation. (**B**) Correlated GO terms plotted as log10(PRNT_80_) vs Normalized GO term DBGGA Bayesian z-scores. The GO term *Viral life cycle* had a negative correlation with seroconversion at the windowed time points –6, –5, and –4 pSN (upper panel), while *Positive regulation of T cell cytokine production* shows a positive correlation with seroconversion at the windowed time points –4, –3, and –2 pSN (lower panel).

Likewise for the Gene Ontology GO terms, we applied the SWC technique to obtain the most highly PRNT_80_ correlated terms for which the results are listed by the sliding window time points (Fig 8). With the more focused gene sets that comprise the GO terms, we observed stronger correlation values and more immune related terms. For example, we detected strong correlation of viral related biological processes such as *viral protein processing* (GO:0019082), *viral life cycle* (GO:0019058) (see correlation plot Fig 8B, upper graph), *viral transcription* (GO:0019083), and *viral release from host cell* (GO:0019076). Additionally, we observed early indicators of the host immune response with the correlation of PRNT_80_ with the biological processes such as *cellular response to interleukin-4* (GO:0071353), *interleukin-1 beta production* (GO:0032611), *negative regulation of B cell activation* (GO:0050869), and *type I interferon signaling pathway* (GO:0060337).

**Fig 9:**
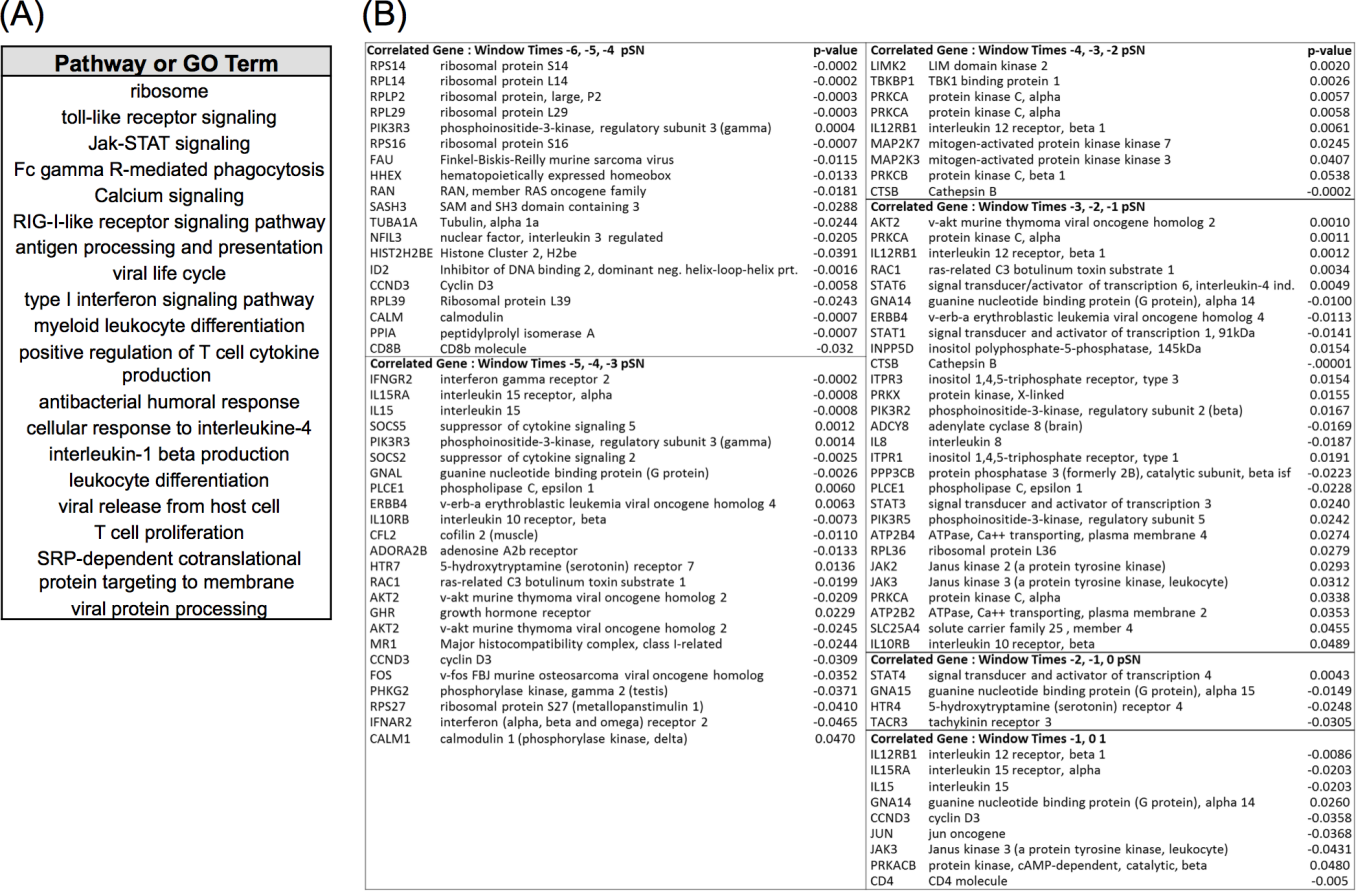
Summary of DBGGA Pathways or GO terms and constituent genes with high correlation to protective antibody response. (**A**) A selected set of DBGGA scored pathways or GO terms most correlated with protective antibody response employed for learning the dynamic Bayesian network model for inferencing future protective immune response. (**B**) A subset of immunologically relevant genes associated with pathways and GO terms listed in (A) that are highly correlative to serologic protection as determined by PRNT_80_. Various time windows are represented, with time –6, –5, –4 pSN window being the furthest from the designated time of serologic protection.

To narrow the search space for genes that were candidate biomarkers of protective immunity, we selected the sets of genes for pathways and GO terms having significant correlations from 19 relevant pathways and processes (Fig 9A). The SWC technique identified 145 unique highly correlated genes as listed in S15 Table. A subset of most highly correlated and relevant genes is listed in the table of Fig 9B. There was a dominance of genes encoding a large family of ribosomal proteins that were overlapping across the biological processes that included *viral life cycle*, *SRP-dependent co-translational protein targeting to membrane*, *viral protein processing*, *viral release from host cell*, and *ribosome pathway*. Accordingly only a few of these gene types were represented in the table. Interestingly, the ribosomal genes were among the highest SWC correlated relationships to occur at the earliest time window (Times –6, –5, –4 pSN). Also of note, many of the genes in the first two time windows are negatively correlated, but interestingly, the genes PIK3R5, SOCS5, PLCE1, and GHR were positively correlated. The majority of genes in the remaining time windows was positively correlated. It was also observed that the later time windows (Times –5, –4, –3 pSN and Times –4, –3, –2 pSN) had a reduced number of correlated genes and weaker significance (p-values) of correlation.

### Dynamic Bayesian Network (DBN) predicts protective immunity

The SWC analysis identified a set of genes associated with certain pathways and GO terms (See Table 1). A set of correlated genes associated with PRNT_80_ response was insufficient on their own for building a predictive model, since it should be constructed based on biologically relevant relationships and cascade of events. There were other relationships both upstream and downstream from these genes that may have critical roles in defining transcriptional events leading to a protective immune response. It was this set of biologically related genes of the innate immune response that are of prime interest, and hence were used to create a DBN model. We employed a two-step approach to constructing a predictive DBN. The first step employed a novel approach to learning a gene regulatory network (GRN) structure that is described in more detail in S1 Text Section 4.0. In this approach, the starting point is to seed the learning algorithms with a starting set of genes, which in our case were the genes associated with perturbed pathways that had the majority of temporal correlated gene expressions to PRNT_80_ found by the SWC analysis. These pathways included the *Toll-like receptor*, *Jak-STAT signaling*, *RIG-I-like signaling*, *Fc gamma R-mediated phagocytosis*, *Calcium signaling*, *Antigen processing and presentation*, *selected ribosomal genes from viral life cycle*, and included genes with type I interferon activation.

**Table 1:** Sliding Window Correlation of Sets of Genes Associated with Pathways and GO Terms.

Immediate innate immune response	Pre-seroconversion triggering adaptive immune response	Pre-seroconversion triggering adaptive immune response	Onset of protective immunity
-6 pSN	-5 pSN	-4, -3, -2 pSN	-1, 0, 1 pSN
GO Term	GO Term	GO Term	Pathways
Defense response to virus	Lymphocyte activation	Viral gene expression	Wnt signaling
Viral gene expression	Leukocyte activation	Viral life cycle	Phosphatidylinositol signaling
Interspecies interaction between organisms	Viral gene expression	Lymphocyte activation	mTOR signaling
Type 1 interferon signaling response to cytokine	Viral life cycle	Leukocyte activation	Jak-STAT signaling
Interferon-gamma-mediated signaling	Leukocyte differentiation	Regulation of apoptotic process	B cell receptor signaling
**Pathways**	B cell activation	Regulation of cell communication	Insulin signaling
Jak-STAT signaling	Tcell activation	Actin cytoskeleton organization	VEGF signaling
Phosphatidylinositol signaling	Leukocyte aggregation	Immune-response-activating cell surface receptor signaling pathway	ErbB signaling
ECM-receptor interaction	**Pathways**	Leukocyte differentiation	T cell receptor signaling
Complement and coagulation	Jak-STAT signaling	Regulation of IkappaB signaling	Regulation of actin cytoskeleton
Chemokine signaling	Phosphatidylinositol signaling	T cell aggregation	Chemokine signaling
Wnt signaling	ErbB signaling	T cell activation	ECM receptor
	PPAR signaling	Positive regulation Wnt signaling pathway	Gap junction
	Calcium signaling	Fc-gamma receptor signaling pathway involved phagocytosis	MAPK signaling
	Chemokine signaling	Leukocyte cell-cell adhesion	Calcium signaling
	Gap junction	Antigen receptor mediated signaling pathway	Focal adhesion
	Wnt signaling	**Pathways**	Natural Killer cell cytotoxicity
	Toll-like receptor signaling	Phosphatidylinositol signaling	Fc gamma R-mediated phagocytosis
	ECM-receptor interaction	Gap junction	Antigen processing and presentation
	Hedgehog signaling	Wnt signaling	
	MAPK signaling	Jak-STAT	
		Chemokine signaling	
		ECM-receptor	
		PPAR signaling	
		Calcium signaling	
		Toll-like receptor signaling	
		Insulin signaling	
		mTOR signaling	
		Adipocytokine signaling	
		Regulation of actin cytoskeleton	
		B cell receptor signaling	
		Ribosome	
		Antigen processing and presentation	

The GRN method used a Bayesian model consensus scheme employing the Markov Chain Monte Carlo (MCMC) Metropolis-Hastings algorithm. It incorporates a gene expression data-derived proposal matrix and *a priori* probability distribution method for combining prior biological knowledge from multiple sources that included KEGG [37], REACTOME [62], BioGRID [8], DIP [63], IntAct [64], MINT [65], GO, and predicted and known transcription factors (TF) binding sites from TRANSFAC [66] and JASPER [67]. For the second step, the output of the GRN provided a unique approach to identifying high probability relationships between the highly correlated genes and other upstream and downstream genes. The GRN identified correlated genes and their relationships to other genes in which the relationships were found through either known canonical relations, predicted based on protein domain binding likelihood, sequence similarity to know binding domains, or through transcription factor binding. Through this technique, we narrowed the choice of DBN model genes to 50 employing a selection method that focused on SWC genes and their upstream and/or downstream GRN learned relationships that resulted in the selection of cell receptors, signaling processing, and gene end products. The complete descriptions of these 50 genes are provided in S9 Table. By interrogating the GRN model for key regulatory relationships, we found strong supporting evidence that upon vaccination, the vaccine RNA could be recognized by the Toll-like Receptors (TLR), or more specifically, TLR4, a negative SWC correlate to PRNT_80_ response. Expression of other specific genes having learned relationships downstream of TLR4 were found to include MYD88, MAP2K7, IL1B, FOS, and JUN, where FOS and JUN form the well-known transcription factor complex AP-1. TLR mediated signaling pathway predominately signal through interferon regulatory factors (IRF) as well as Nuclear Factor-kappa B (NFKB) and AP-1, eliciting the induction of the Interferon type-1 response and the expression of inflammatory cytokines. In support of interferon activation, GRN learning identified IFNAR1/2 and IFNGR1/2 as influential regulators in the GRN model. These genes encode proteins that function as antiviral factors. We found IFNAR2 to be a positive SWC correlate to PRNT_80_, and through our GRN learning, we found evidence supporting an activation relationship with STAT1 and a STAT1 relationship with the cytokines CISH and SOCS2.

In addition, we found other type I interferon associated GRN model genes that included MYD88, JAK2, SOCS2/5, CCL5, IL8, IL15, IL12RB1, and IRF9. The genes MYD88, SOCS2, IL8 and IL15 were negative SWC correlates to PRNT_80_ response while IL12RB1, JAK2 and SOCS5 were positive correlates. Also found by GRN learning was the gene, SYK, which is critical to innate and adaptive immunity having strong evidence regulating the downstream gene, PIK3R3, which is a positive SWC correlate to PRNT_80_ response. Also, the RIG-I-like receptor related genes, IFIH1 and DDX58, were identified by the GRN model as strong regulators. However, these genes were not found to be SWC correlates, but were significantly expressed and indirectly linked to other SWC correlate genes such as TBKBP1 and IL8. IFIH1 and DDX58 genes are known to encode for proteins of the RIG-I-like receptor family and functions as pattern recognition receptors that sense viral nucleic acids or other viral/vaccine products.

The genes ERBB4 and ADORA2B (positive and negative SWC correlates to PRNT_80_ respectively) were found to be important receptors and perhaps novel to the correlated response to RVF vaccine. ERBB4 encodes a protein that is a receptor for neuregulins and EGF family members and is known to regulate cell proliferation, differentiation, migration and apoptosis. ADORA2B encodes a G-protein receptor and is known to inhibit monocyte and macrophage functions and stimulate mast cell mediator release. GRN learning found ERBB4 to have strong relationships with PLCB1 and PLCE1 that is a positive SWC correlate. PLCE1 belongs to the phospholipase family involved in the cascade of intracellular responses that result in cell growth and differentiation. GRN learning associated ADORA2B with the downstream genes GNAL, ADCY7, PRKX and ATP2B2 are positive SWC correlates to PRNT_80_ response suggesting a novel role as correlates to protective immunity. Vaccine viral replication and expression were strongly indicated in the early phase post immunization. Numerous genes encoding for ribosomal proteins were SWC correlates to PRNT_80_. We chose four of the most highly correlated to include in the DBN model, namely, RPS14, RPL14, RPL29 and RPLP2.

In the later times (Times –4, –3, –2, –1, 0 pSN) where the time begins to overlap with the onset of serologic protective response, there were fewer SWC correlates to PRNT_80_. Interestingly, antigen processing and presentation stood out to have several later phase PRNT_80_ correlates with the genes CD4, CD8B, CTSB. CD4 is a T-cell surface glycoprotein on T lymphocytes and also in B cells and macrophages. CD4 is thought to be associated with T-cell activation. GRN learning found relationships with three forms of Major Histocompatibility Complex, class II genes, namely, BOLA-DQB, BOLA-DQA1, and BOLA-DRB3, all are expressed on antigen presenting cells (B lymphocytes, dendritic cells, macrophages). CD8B is a CD8 antigen found on most T lymphocytes. CTSB is a gene encoding a lysomomal cysteine proteinase believed to participate in intracellular degradation of proteins. The description of these genes and function is provided in S16 Table.

**Fig 10:**
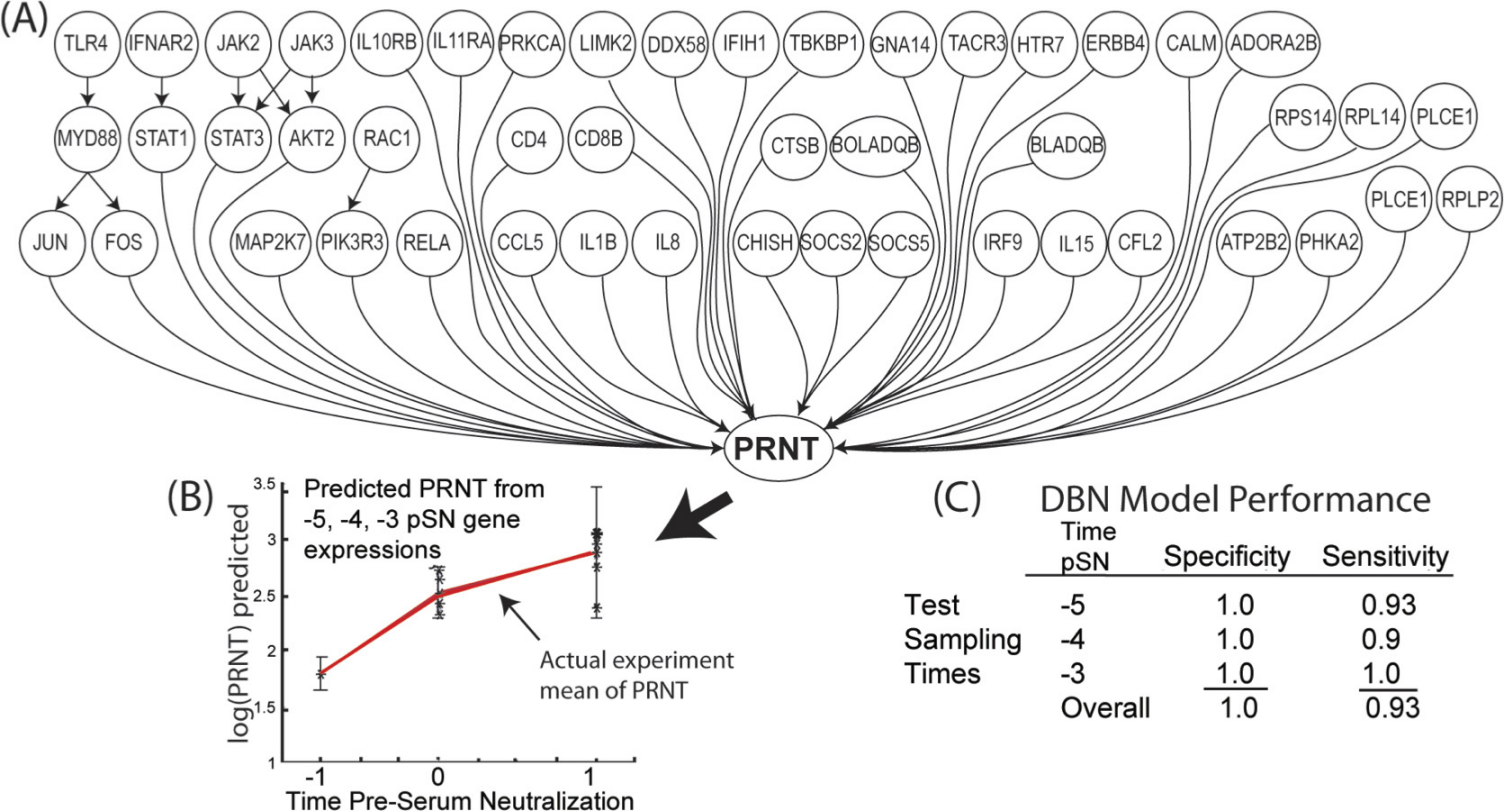
Prediction of protective immunity by the Dynamic Bayesian Network (DBN). (**A**) The Dynamic Bayesian network model representing the learned gene regulatory network structure. Each node represents a continuous variable with a Gaussian distribution. The DBN was trained with the time course data associated with each gene. Relationships between gene nodes, or between the gene node and the PRNT_80_ node, are represented by arrows as a directed acyclic graph. (**B**) Example inferencing results on a selected set of gene expression data taken at time window -5, -4, -3 pSN to predict the PRNT_80_ outcome. Graph indicates strong serologic response and log10(PRNT_80_) levels sufficient for protection given hypothetical gene expression data. (**C**) Performance table for model experiment in (B) show high specificity and sensitivity of the model predictions in a time window up taken at –5, -4, -3pSN. Data sets for true negative testing were randomly selected from other genes at the same time points as the true positive gene data sets. Data from five individual animal subjects were employed for the model evaluation.

Employing the majority of these genes described above, the DBN model was constructed to represent three early phase time points (T = –5, –4, and –3) pSN, but included the later PRNT_80_ data time points (T = –1, –0, and 1 pSN) for training the model to be predictive for these later PRNT_80_ time point responses. The DBN network is illustrated in Fig 10A. Each gene and PRNT_80_ node represents a continuous variable having Gaussian distribution.

The goal of the DBN model was to determine if gene expression evidence at earlier time points could robustly predict/infer a protective PRNT_80_ response at later time points. With limited sample size, we could only train and test the predictive response with gene expression evidence from five biological replicates at three distinct time points (Times –5, –4, and –3 pSN). Cross-validation and blind prediction were not feasible and are planned for future research validation efforts. We implemented a *t*-test statistic to test when an inferred PRNT_80_ value was determined to be statistically outside the experimental (true) mean population (p ≤ 0.05) for use in sensitivity analysis. Expression data for the true gene sets were used for the true positive test cases which consisted of individual time point data sets of the three time points and five biological replicates. A randomly selected set of gene expressions were used to create a test set to represent the true negative test cases. The predicted PRNT_80_ plot, Fig 10B, and model performance table, Fig 10C, illustrates the robustness of the resulting model.

## Conclusions

Understanding the mechanisms underlying the development of protective immunity against RVFV, and RVF vaccination, will be important for the development of more effective vaccines against RVF. Host genome-wide transcript abundance and signaling pathway profiling provides a means to identify changes in gene expression and biological function occurring immediately following vaccination that may play a role in the development of protective immunity. Here, we used the hypothesis that bioinformatic analysis of deeply sequenced peripheral blood mononuclear leukocytes (PMBC) transcript will identify highly correlated gene transcripts for protective seroconversion as measured by PRNT_80_. This approach has revealed transcriptomic signatures underlying the host response to vaccination with arMP-12, as well as a distinct set of genes that would be expected to be predictive of a protective immunological response.

There were 1587 unique genes differentially regulated following immunization by arMP-12, of which 678 were uniquely up regulated genes compared to 909 uniquely down regulated. Consistent with other previously reported gene expression results in the mouse model infected with RVFV [68], the larger number of down regulated genes is likely a reflection of the high amount of arMP-12 vaccine NSs that is able to inhibit the transcription activity of constitutive promoters [61, 69]. Germline-encoded receptors (e.g. TLRs, RIG-I-like) recognize viral nucleic acids of the vaccine. Complex networks of signal transduction converge on multiple transcription factors that included AP-1, STAT1/3/6, and IRF9. The interplay of these transcription factors determines the transcriptional response profile of downstream genes and leads to a dynamic expression of genes that produce a cascade of biological responses necessary to initiate and achieve long-term immune protection to RVFV.

Immediately following vaccine inoculation, it appears that the vaccine viral components have successfully invaded the host cells, triggering the induction of a family of pleiotropic cytokines known as the IFNs (interferons) as evidenced by the activation of the type I interferon signaling and the significant perturbation of the interferon stimulated genes (ISGs) IFI16, MX1, IFIH1, IFI44, IFNAR2, IFITM1/2, IRF2BP2, IR12RB1, IRF1, IRF3 and IRF9 within the first 5 days post immunization. IFN interactions with their receptors induce a set of IFN-stimulated genes that inhibit viral replication and increase the lytic potential of natural killer (NK) cells. It has been reported [70] that Type-I IFNs modulate the adaptive immune response by increasing MHC-I or MHC-II (Major Histocompatibility Complex Class-I/II) expression to promote antigen presentation, also promoting T-Cell survival and stimulating dendritic cell maturation. However, in our study, the MHC-I genes were only minimally expressed and the only significantly expressed MHC gene was HLA-DOA, (MHC class II) and the MHC-II transactivator gene, CIITA. The gene, CIITA, encodes a protein essential for transcriptional activity of the HLA-II promoter. This protein is located in the nucleus and acts as a positive regulator of MHC-II transcription. This gene has been functionally associated with cellular response to exogenous dsRNA and interferon-gamma-mediated signaling pathway.

At later time points, we observed the triggering/activation of T cells and B cells, suggesting that cell-mediated immunity was beginning as early as day 3 post vaccination. A number of genes were expressed that are associated with T cell activation and signaling that included PIK3R1, PIK3R3, CHUK, ICOS, NFkappaB2, RNUX2, AKT2, CCR2, CD48, RELA, STAT5A, TNFSF14, TLR4, CD72, ITFG2, NR1D1, and PTPN6. Interestingly, the majority of these genes was significantly modulated at days 5 and 6 and became less expressed at day 7 and beyond. Complementary to our work, a recent study using a deletion mutant of arMP-12, arMP12-deltaNSm21/384, showed rapid progression of protective antibodies after vaccination and after challenge with the wild-type strain ZH501 [19]. This rapid antibody response to wild-type challenge was concomitant with INF-γ expression, indicating a cell-mediated immune response. Our data indicate that the parent arMP-12 strain is capable of inducing the adaptive immune response machinery quickly after vaccination.

The primary objective of this retrospective study was to identify a set of genes for predicting protective immunity. The selection of the genes was based on a systems biology top-down approach in which we narrowed our focus on genes within pathways and GO terms that were significantly perturbed and had SWC correlates to the PRNT_80_ onset of protective immunity. Novel in this approach was the fact that we employed a sliding time window to find trajectory responses at earlier time points that have high positive or negative correlation to the PRNT_80_ response trajectory at time points just prior to and at the point of protective seroconversion.

An additional novel step was then employed to learn the gene regulatory network (GRN) from the selected set of genes. This step allowed us to further identify genes that had strong regulatory relationships among other upstream and downstream genes. Finally, the combination of SWC correlates to PRNT_80_ and the GRN relationships were used to select the genes used in constructing and training the predictive DBN model. The model performance was quite good when the model was tested with gene expression profiles from Time -5, -4, or -3 pSN and was successful in predicting a future PRNT_80_ value at Time –1, –0, and 1 pSN. In summary, the model had an overall sensitivity = 93% and specificity = 100%. While the limited number of replicates restricts our ability to fully test the robustness of this model, it does allow us to access our approach to developing models predicting protective immunity.

## Supporting Information

S1 Table Total number of reads, read alignments, and mapped reads by animal and time point. (XLSX)

S2 Table Top 15 Up or Down Regulated Genes by Day Post Inoculation (PI). (XLSX)

S3 Table List of significantly scored genes by days post immunization using DESeq. (XLSX)

S4 Table List of significantly scored Pathways by days post immunization using DBGGA. (XLSX)

S5 Table List of genes from significantly scored Pathways by days post immunization using DBGGA. (XLSX)

S6 Table List of significantly scored GO Terms by days post immunization using DBGGA. (XLSX)

S7 Table List of genes associated with significantly scored GO Terms by days post immunization using DBGGA. (XLSX)

S8 Table Top 15 Up or Down Regulated Genes by time pre-Seroconversion (pSN). (XLSX)

S9 Table List of significantly scored genes by time pre-seroconversion (pSN) using DESeq. (XLSX)

S10 Table Common Genes from Differential Gene Expression Data: Standard Time vs. Time Shifted. (XLSX)

S11 Table List of significantly scored Pathways by time pre-serum neutralization using DBGGA. (XLSX)

S12 Table List of genes from significantly scored Pathways by time pre-serum neutralization using DBGGA. (XLSX)

S13 Table List of significantly scored GO Terms by time pre-serum neutralization using DBGGA. (XLSX)

S14 Table List of genes from significantly scored GO Terms by time pre-serum neutralization using DBGGA. (XLSX)

S15 Table Excel spread sheet with multiple workbooks showing results for the Sliding Window Correlation analysis of pathways, gene ontology terms, and their associated genes. (XLSX)

S16 Table Predictive genes for immunologic protection as generated by Dynamic Bayesian Network Analysis. (XLSX)

S17 Table DBGGA vs GAGE Kolmogorov-Smirnov Overlapping Pathways by time pre-serum neutralization. (XLSX)

S18 Table Pathway Scoring by GAGE GSEA t-test method by time pre-serum neutralization. (XLSX)

S19 Table DBGGA vs GAGE GSEA t-test Overlapping Gene Ontology Terms by time pre-serum neutralization. (XLSX)

S20 Table Gene Ontology GSEA Results Employing GAGE t-test method by time pre-serum neutralization. (XLSX)

S1 Text (DOCX)

S2 Text (DOCX)
