# Correction: Correlative Gene Expression to Protective Seroconversion In Rift Valley Vaccinates

**DOI:** 10.1371/journal.pone.0156469

**Published:** 2016-05-23

**Authors:** Richard C. Laughin, Kenneth L. Drake, John C. Morrill, L. Garry Adams

Figs 6 and 7 are duplicates of Fig 5. Please view the correct Figs 6 and 7 below.

**Fig 6:**
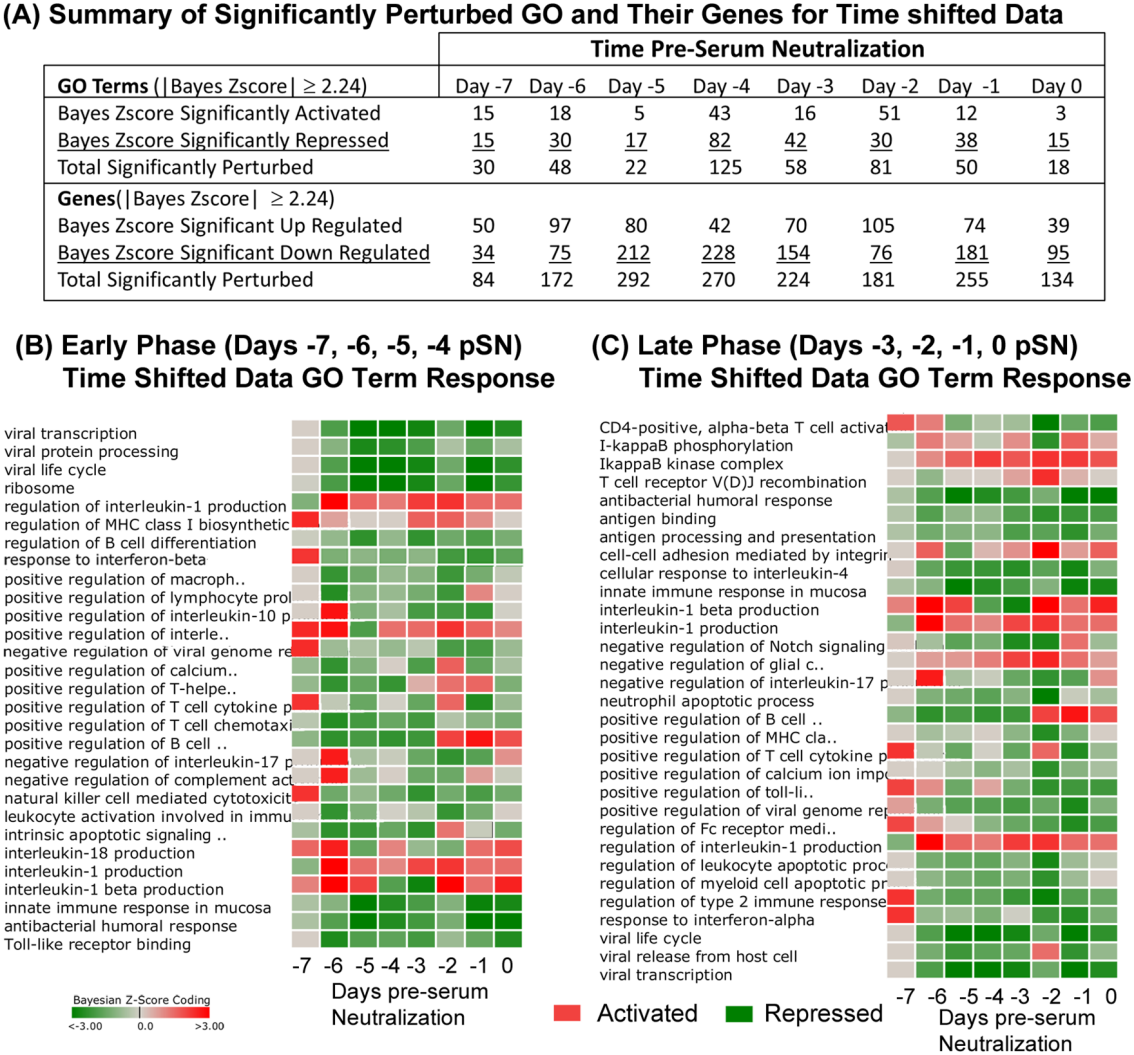
DBGGA GO term analysis on time-shifted data. (**A**) Summary table of GO Terms and component gene perturbation by time pre-seroconversion. Only GO terms and their genes with Bayesian score ≥|2.24| are included in analysis. (**B-C**) Heat maps of perturbed GO terms described by time pre-seroconversion and identified from the Early Phase (time –6, –5, –4, –3 pSN) (**B**) and the Later Phase (time –2, –1, 0, 1 pSN) (**C**). Red color indicates activation, green color indicates repression. Intensity of color represents amplitude of perturbation. The list of GO terms shown represent a subset of all perturbed terms that were selected as being most relevant to innate and adaptive immune response.

**Fig 7:**
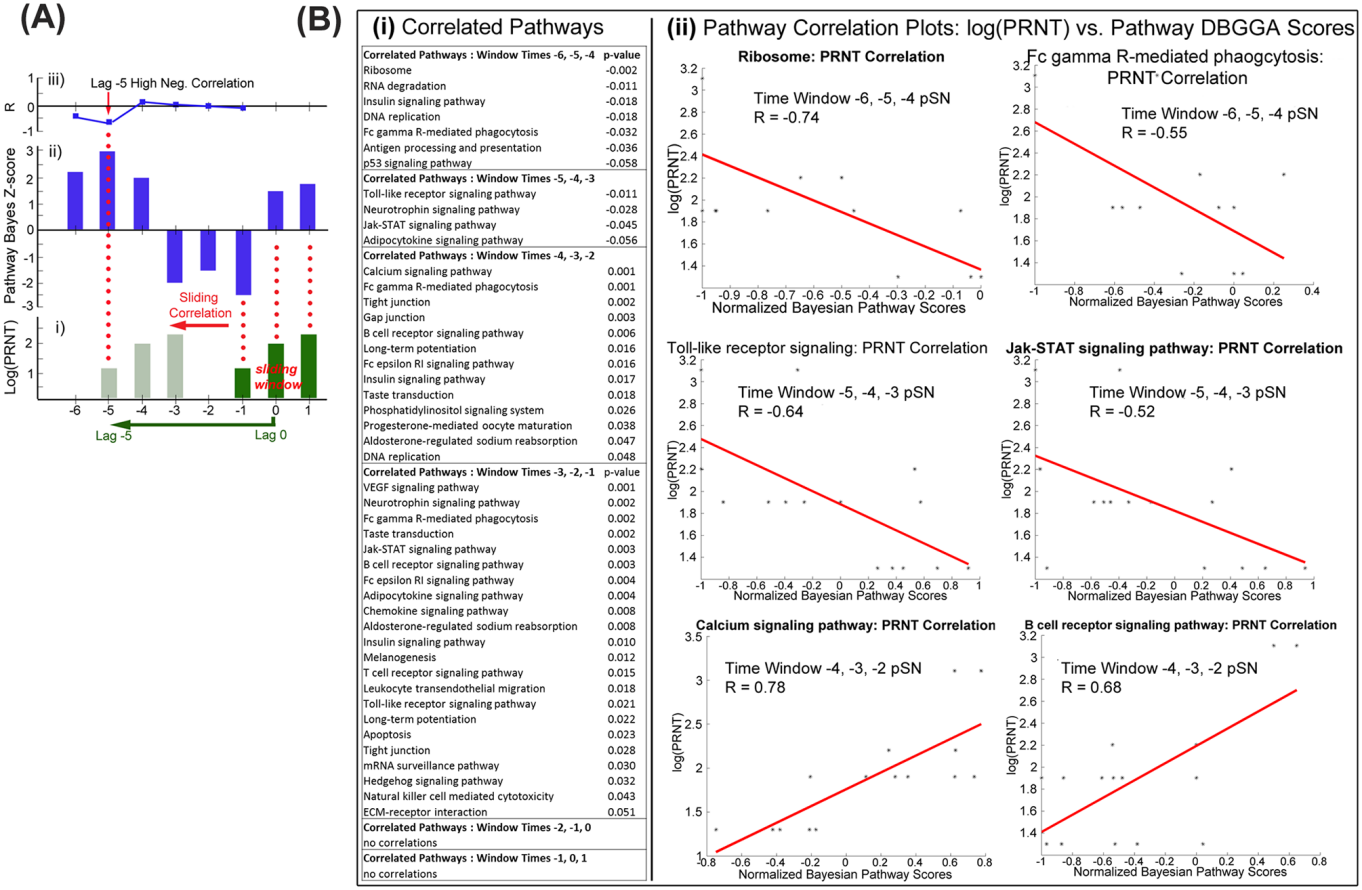
Sliding window correlation (SWC) to identify pathways associated with serum neutralization titers. (**A**) Visualization of sliding window correlation approach. (**i**) Hypothetical Log10(PRNT_80_) data taken from time points capturing neutralizing antibody levels during time period in which animals reached threshold for protection (log10 = 1.903) (dark green bars). (**i-ii**)The trajectory of the Log10(PRNT_80_) data is applied to other time points prior to serum neutralization (light green bars) to identify pathway with complementary or antithetical trajectories (**A-ii** for hypothetical Pathway Bayesian Z-score data, dark blue bars). (**iii**) Graphical representation of the R correlation coefficient value between Log10(PRNT_80_) and Pathway Bayesian Z-score. (**B**) Pathways correlated to PRNT_80_values at the time of seroconversion listed by pathway at incremented window times (each consisting of three time points), with significant correlation p-values (**i**), or plotted as log(PRNT_80_) vs normalized Bayesian Z-score (**ii**) for six selected pathways having highest correlations.
