# Supplementary material for: Correlative Gene Expression to Protective Seroconversion in Rift Valley Fever Vaccinates
Source: PLoS One. 2016 Jan 19;11(1):e0147027. doi: 10.1371/journal.pone.0147027 (PMC4718665; doi:10.1371/journal.pone.0147027)
Supplement: S2 Text — (DOCX) [file pone.0147027.s022.docx]

**S2 Text**

**1.0 Pathway Analysis Alternative Comparison**

This section includes comparative results between the DBGGA pathway scoring method and an alternate gene set enrichment analysis (GSEA) approach provided by the R package “GAGE” [[1](#_ENREF_1)]. As would be expected, there is some overlap between the two approaches in pathways having a large number of highly expressed genes. Table 1 illustrates the overlap for each time point based on the sliding window correlation (SWC) times. The comparison is limited to the SWC datasets since the manuscripts conclusions are based on this time shifted data.

| **Table 1. Number of Significantly Perturbed Pathways by SWC Time Comparison to DBGGA to GAGE** | | | | | | | | | |
| --- | --- | --- | --- | --- | --- | --- | --- | --- | --- |
|  | time -6 | time -5 | time -4 | time -3 | time -2 | time -1 | time 0 | time 1 |  |
| **DBGGA** | 6 | 18 | 20 | 21 | 32 | 23 | 38 | 11 | DBGGA \|z-score\|>2.24 |
| **GAGE GSEA t-test method** | 4 | 6 | 7 | 7 | 8 | 10 | 8 | 8 | p-value < .025 |
| **# Overlap with DBGGA** | 0 | 3 | 3 | 3 | 5 | 6 | 6 | 3 |  |

The actual overlapping pathways are listed in S17 Table. The differences in the overlapping list appears to be related to the larger number of pathways and gene sets that were scored by the GAGE method and how the genes are ranked for application in their tests. However, the overlap does offer very high confidence that these pathways are significantly modulated by the RVF vaccine. For example the Ribosome, Gap junction, Calcium signaling, and Chemokine signaling pathways are all dominate pathways at multiple time points in both the DBGGA and GAGE methods.

The S18 Table includes the GAGE based p-value and q-value scores for all pathways scored by the GAGE Kolmogorov-Smirnov test. The GAGE t-test method was excluded since it provided similar overlaps to the GAGE Kolmogorov-Smirnov test.

**2.0 Alternative Gene ontology enrichment results**

This section includes comparative results between the DBGGA GO scoring mechanisms and an alternate gene set enrichment analysis (GSEA) approach provided by the R package “GAGE” [[1](#_ENREF_1)]. As would be expected, there is some overlap between the two approaches in GO terms, but the percentage of overlap appears significantly less than for the pathway comparisons (Table 2). Table 2 illustrates the number of overlaps for each sliding window correlation (SWC) time point. As in the above pathway comparison, the analysis is limited to the comparison of the SWC datasets since the manuscripts conclusions are based on this time shifted data. The main issue between the comparison differences is the way in which the two approaches select the GO term gene sets. In the GAGE GO scoring, the gene sets were limited to “biological processes” only, while for DBGGA the analysis included “molecular function” and “cellular location”. Accordingly, there were several more GO groups found significant in the DBGGA results. This will also create more redundant scoring, since there are many genes that overlap across the three broad GO categories. The actual overlapping pathways are listed in S19 Table.

| **Table 2. Number of Significantly Perturbed Gene Ontology Terms by SWC Time Comparison: DBGGA to GAGE** | | | | | | | | | |
| --- | --- | --- | --- | --- | --- | --- | --- | --- | --- |
|  | time -6 | time -5 | time -4 | time -3 | time -2 | time -1 | time 0 | time 1 |  |
| **DBGGA** | 0 | 90 | 97 | 124 | 165 | 99 | 158 | 48 | (DBGGA \|z-score\|>2.24 |
|  |  |  |  |  |  |  |  |  |  |
| **GAGE GSEA t-test method** | 0 | 7 | 13 | 8 | 11 | 7 | 12 | 10 | p-value < .025 |
| **Number Overlap with DBGGA** | 0 | 0 | 7 | 4 | 2 | 0 | 6 | 3 |  |

The S20 Table includes the GAGE based p-value and q-value scores for all GO terms scored by the GAGE GSEA t-test method. The most striking difference between the DBGGA approach and GAGE was that GAGE did not identify any viral related processes as was found by the DBGGA method. DBGGA found several viral related GO terms highly perturbed which included, for example, “virion assembly”, “viral transcription”, “viral release from host cell”, “viral protein processing”, and “viral life cycle”. Furthermore, GAGE GSEA did not identify other important processes known to be associated with vaccination response or a response to viral proteins such as “response to interferon-alpha”, “positive regulation of interferon-alpha production” or any important immune response processes such as “CD4-postive, alpha-beta T cell activation or regulation of type 2 immune response, which were all found by the DBGGA method.

1. Luo, W., et al., *GAGE: generally applicable gene set enrichment for pathway analysis.* BMC Bioinformatics, 2009. **10**: p. 161.
